# Case Report: A neoantigen-targeting peptide vaccine combined with checkpoint inhibition induces tumor regression and long-term remission in a pediatric patient with metastatic hepatocellular carcinoma

**DOI:** 10.3389/fimmu.2025.1674663

**Published:** 2025-10-31

**Authors:** Germano Amorelli, Armin Rabsteyn, Claus-Philipp Maier, Finn Trautner, Ursula Holzer, Jürgen Frank Schäfer, Martin Ebinger, Rupert Handgretinger, Sven Nahnsen, Hans-Georg Rammensee, Peter Lang

**Affiliations:** ^1^ Department of Hematology, Oncology, Gastroenterology, Nephrology, Rheumatology, University Children’s Hospital, Tuebingen, Germany; ^2^ University of Tübingen, German Cancer Consortium (DKTK) and German Cancer Research Center (DKFZ), Partner Site Tübingen, Tuebingen, Germany; ^3^ Department of Hematology, Oncology, Clinical Immunology and Rheumatology, Center for Internal Medicine, University Hospital Tuebingen, Tuebingen, Germany; ^4^ Department for Diagnostic and Interventional Radiology, University Hospital, Tuebingen, Germany; ^5^ Quantitative Biology Center (QBiC) Tübingen, University of Tübingen, Tübingen, Germany; ^6^ Institute of Immunology, University of Tübingen, Tübingen, Germany; ^7^ Cluster of Excellence iFIT (EXC 2180) Image-Guided and Functionally Instructed Tumor Therapies, University of Tuebingen, Tuebingen, Germany

**Keywords:** pediatric hepatocellular carcinoma, peptide vaccination, immune checkpoint inhibition, nivolumab, neoantigen-specific T-cells

## Abstract

Pediatric hepatocellular carcinoma (HCC) is a rare and aggressive malignancy with limited treatment options and poor prognosis, highlighting the need for innovative therapeutic strategies. Neoantigen-targeting peptide vaccination is a promising treatment approach with potential for combination therapy with checkpoint inhibition (CPI). Here, we present a case study of a pediatric patient with metastatic HCC treated with a neoantigen-derived peptide vaccine combined with CPI therapy after disease recurrence. Immunomonitoring revealed robust vaccine-induced T-cell responses, further enhanced by CPI. T-cell cloning and T-cell receptor (TCR) sequencing confirmed neoepitope specificity and clonality of the vaccine-induced T-cell response. Following immunotherapy, the inoperable metastasis regressed completely, with no further intervention. A subsequent metastasis was surgically resected, and the patient has remained in complete remission since, with an overall survival (OS) of 13 years. These findings underscore the potential of personalized peptide vaccination and demonstrate the feasibility and efficacy of combinatorial strategies in optimizing therapeutic outcome in pediatric HCC. Importantly, this case illustrates a uniquely durable remission in pediatric metastatic HCC, exceeding survival outcomes reported in previous vaccine or CPI monotherapy studies.

## Introduction

Pediatric HCC, though rare, is aggressive and the second most common primary liver malignancy in children after hepatoblastoma, with a 5-year survival rate of 46.4% (1, 2). CPIs show promise in enhancing anti-tumor responses in HCC (3), though not all patients benefit, highlighting the need for novel approaches (4).

In order to improve the therapeutic efficacy of CPI monotherapy, various combination strategies are currently explored in advanced HCC (5). These include dual CPI regimens (e.g., CTLA-4 and PD-L1 inhibitors) (6), combinations with anti-angiogenic agents (e.g., PD-1 and VEGF inhibitors), and regimens incorporating multi-target tyrosine kinase inhibitors (TKIs). Nevertheless, CPI-resistant HCC continues to represent a significant clinical challenge (7). Thus, alternative therapeutic approaches, such as personalized peptide-based vaccinations, warrant rigorous evaluation with regard to their safety and efficacy (5).

This case involves a pediatric patient with HCC and hereditary fructose intolerance (HFI), a rare metabolic disorder caused by aldolase B deficiency (8), potentially contributing to oncogenesis. Tumor mutational burden (TMB) has been proposed as a biomarker for progression and treatment response, with high TMB correlating with reduced survival but potentially better CPI efficacy (9, 10).

Neoantigens arising from cancer mutations enable T-cell recognition, making personalized peptide vaccination a promising strategy. Peptide-based vaccines, targeting HLA class I and II molecules, have shown progress in eliciting CD4^+^ and CD8^+^ T-cell responses (11–13).

We report a pediatric HCC patient with refractory disease and inoperable metastases achieving complete, durable remission after neoantigen-targeting peptide vaccination combined with Nivolumab.

## Case presentation

In 2011, a 16-year-old female adolescent was diagnosed with grade IV metastatic HCC. At the time of initial diagnosis, the patient presented with pain localized to the region of the left costal margins, accompanied by B symptoms. Cross-sectional imaging showed an extensive mass lesion (7.7 × 6.9 cm) in the left liver lobe, additional lesions in segments 4a (1.8 × 1.9 cm), 4b (3.0 × 1.7 cm), 7 (1.9 × 1.4 cm), and one in the liver hilum (5.8 × 4.4 cm). In the region of the posterior twelfth rib, an osteodestructive lesion (5.1 × 2.7 cm) was detected. Furthermore, a large lymph node metastasis (4.1 × 2.4 cm) exerted a mass effect on the pancreas. Definitive diagnosis was established through biopsy of the costal lesion. Neoplastic cells demonstrated immunohistochemical positivity for HepPar1, Glypican 3, Cam 5.2, and AE1/3, while staining negative for Desmin, Myogenin, and S100, thereby confirming the diagnosis of HCC. Laboratory analysis revealed no elevation in serum concentrations of alpha-fetoprotein (AFP) and carbohydrate antigen 19-9 (CA19-9).

Treatment included neoadjuvant chemotherapy with Cisplatin and Doxorubicin in November 2011, followed by Sorafenib. Surgery in December 2011 comprised left hemihepatectomy, partial diaphragmic resection, and removal of paravertebral metastases.

A paravertebral relapse at T12 was diagnosed in October 2012 and treated with photon radiotherapy in early 2013. A second relapse arising from residual mass occurred in September 2015. Peptide vaccination was initiated in July 2016 on a compassionate use basis at the University Children’s Hospital of Tuebingen. The prime-boost protocol comprised 19 intradermal vaccinations over two cycles, using Sargramostim and Imiquimod as adjuvants.

In November 2016, a left lung metastasis was resected. From March 2017, Nivolumab was added starting with the 14th vaccination and administered at three-week intervals until February 2019. FDG PET-MRI in April 2017 showed persistent activity, while CT in July 2017 revealed significant regression, with complete remission confirmed by October 2017.

A second vaccination cycle, initiated in January 2018 to boost vaccine-induced immunity, was discontinued in March due to an allergic reaction accompanied by a generalized exanthematous rash, urticaria and nausea. Management included intravenous administration of corticosteroids and dimetindene. Under this treatment, the patient recovered fully without sequelae. Vaccine-related side effects were otherwise mild and included local swelling, redness, and pain at the injection site not requiring treatment. No severe (CTCAE grade ≥ 3) adverse events occurred.

**Figure 1:**
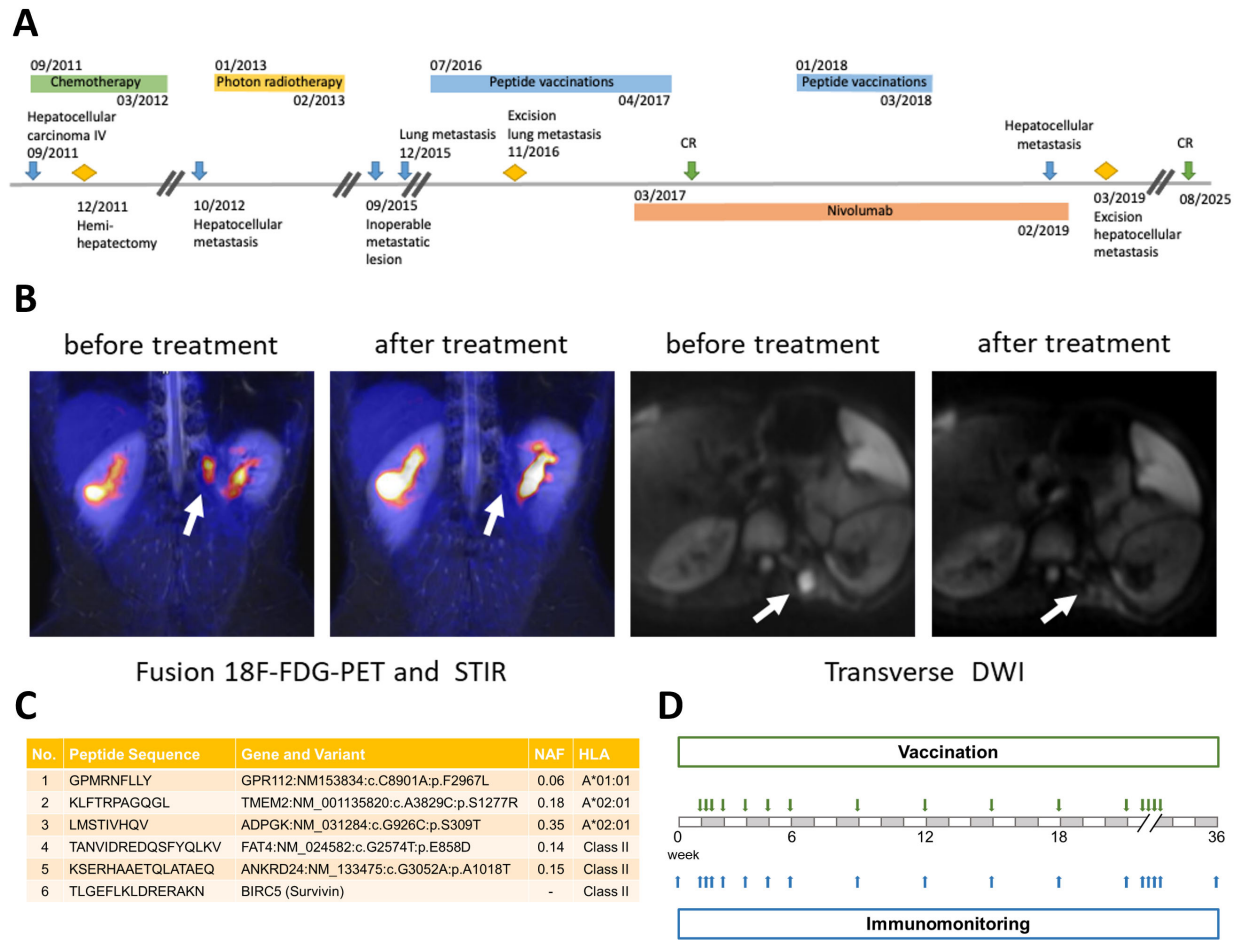
Overview of clinical course, representative imaging, peptide selection and vaccination/immunomonitoring schedule: **(A)** Timeline of the patient’s clinical course, including initial diagnosis, relapses, surgical interventions, and immunotherapy. Blue arrows indicate key events related to the malignancy: initial HCC diagnosis, first relapse (paravertebral metastasis), second relapse (local recurrence arising from residual tumor mass), third relapse (lung metastasis), and fourth relapse (paravertebral recurrence in the *M.erector* sp*inae*). Yellow diamonds mark surgical interventions: initial hemi-hepatectomy, partial diaphragmic resection, and resection of the paravertebral lesion. Green arrows indicate complete remission (CR). Treatment periods are represented by colored bars: chemotherapy (green), photon radiotherapy (yellow), peptide vaccination (blue), and Nivolumab therapy (orange). **(B)** Imaging before and after treatment. Left: fusion 18F-FDG-PET and short tau inversion recovery (STIR) images. Right: transverse diffusion-weighted imaging (DWI). White arrows highlight tumor sites. Imaging demonstrates reduced metabolic activity and resolution of lesions following therapy. **(C)** Selected peptides for the vaccine cocktail, with corresponding peptide sequences, associated genes and variants, novel allele frequencies (NAF), and HLA restrictions. Five peptides were derived from tumor-specific mutations; a sixth peptide, derived from the wild-type antigen Survivin, served as control. **(D)** Schedule of peptide vaccinations (green arrows) and blood sampling for immunomonitoring (blue arrows) during the first vaccination cycle.

Prior to the first vaccination cycle, PET-MRI had confirmed an FDG-positive, inoperable paravertebral lesion. The combination of vaccination and Nivolumab resulted in complete remission, as confirmed by imaging (**Figure 1B**).

In January 2019, a recurrent paravertebral lesion was detected and resected in March. Immunohistochemistry revealed PD-L1 expression in both paravertebral and lung metastases (2016), suggesting CPI sensitivity. Since 2020, whole-body MRI, performed every six months, has shown a sustained complete remission, most recently confirmed at last follow up in August 2025, with the patient achieving almost 14 years of overall survival (OS). Clinical data were retrospectively collected with written informed consent.

## Vaccine design and vaccination

Comprehensive exome sequencing of tumor and matched blood samples revealed a TMB of 3.8 Var/Mbp and identified 49 somatic missense variants. From 171 potential HLA class I-binding peptides and 49 HLA class II candidates, three short and two long peptides were selected and synthesized via solid-phase peptide synthesis (SPPS) at ≥95% purity. Tumor DNA was obtained from a metastatic specimen, while germline DNA was derived from blood. Candidate peptides were ranked according to predicted HLA binding affinity (IC50< 500 nM, netMHC-3.0, netMHCpan-2.4, SYFPEITHI with ≥ 50% of the maximum score), tumor gene expression (Human Protein Atlas), variant allele frequency, and biological relevance of the affected gene. Peptides that were identical to a naturally occurring human wild-type sequence (UniProtKB/Swiss-Prot, human, 9/7/14), or predicted to be poorly soluble due to cysteine content or hydrophobicity, were excluded. Peptides fulfilling these thresholds and derived from tumor-specific mutations were prioritized for synthesis.

The final vaccine cocktail, formulated in 33% DMSO/H_2_O, contained six synthetic peptides (1 mg/ml): five targeting tumor-specific mutations and one control peptide derived from the wild-type antigen Survivin (**Figure 1C**).

Vaccinations were administered intradermally as 300 µl of peptide cocktail (300 µg/peptide), accompanied by subcutaneous Sargramostim (GM-CSF, 1µg/kg body weight) prior to injection, and topical Imiquimod (Aldara cream), applied 30 minutes before and again 24 hours after each dose. The schedule comprised three initial doses (days 1-3), followed by four weekly, and subsequently four-weekly vaccinations, with 16 doses completed by day 225.

PBMCs were analyzed for T-cell responses by intracellular cytokine staining (ICS) and flow cytometry (**Figure 1D**). Given the tumor’s chemo-refractory nature and limited treatment options, CPI therapy – not yet standard first-line – was initiated as an experimental approach guided by 15% PD-L1 expression in the resected lung metastasis (November 2016). The combination of Nivolumab and personalized peptide vaccination resulted in tumor regression and complete remission while enhancing vaccine-specific T-cell responses. Despite an allergic reaction during the second cycle, immunomonitoring indicated favorable T-cell responses.

## Comprehensive immunomonitoring and therapeutic outcome

To address chemotherapeutic effects, T-cell responses to viral recall antigens were monitored. Robust virus-specific responses indicated preserved immune competence and supported the *de novo* nature of vaccine-induced responses, excluding pre-existing peptide-specific reactivity due to PBMC impairment (data not shown).

**Figure 2:**
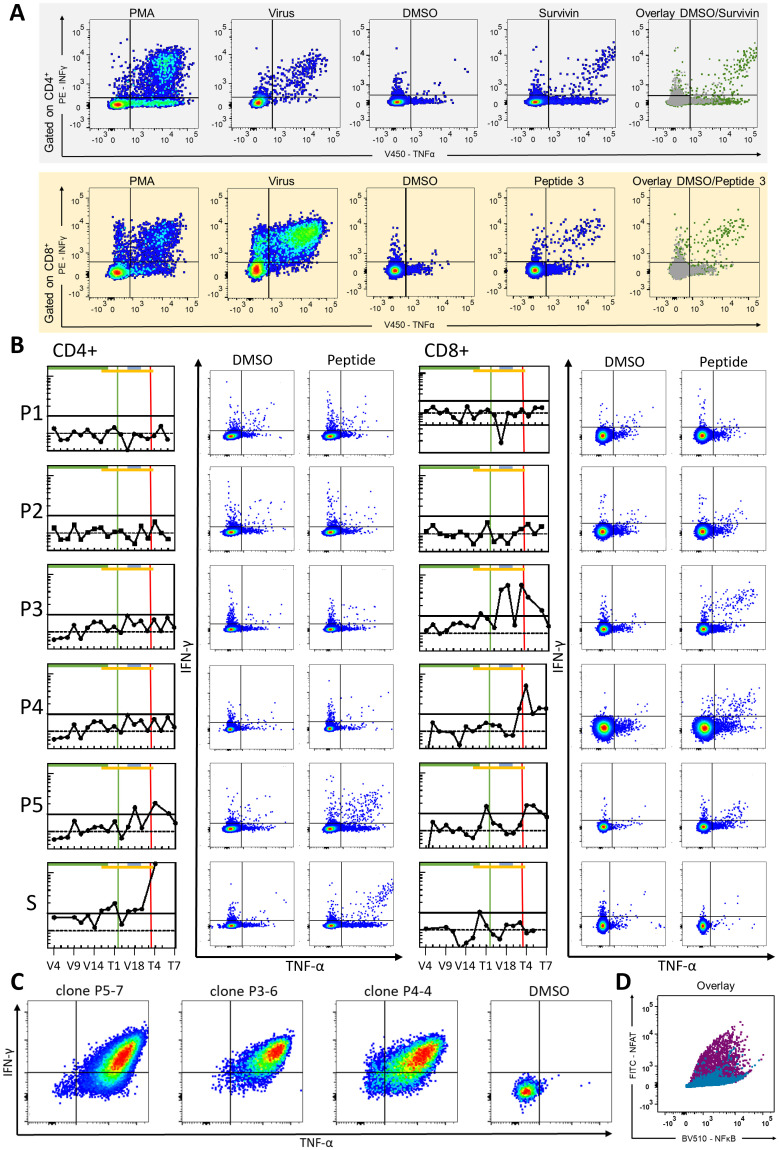
Gating strategy, vaccine-specific t-cell responses and T-cell clone evaluation and TCR validation: **(A)** Representative FACS plots of CD4+ (upper row) and CD8+ (lower row) T-cell responses displaying IFN-γ (y-axis) and TNF-α (x-axis) expression. Sample columns, from left to right, display the following: positive control (PMA/Ionomycin), recall antigen (virus), negative control (DMSO), peptide-stimulated (peptide 3), and overlay of the negative control (gray) and peptide-stimulated sample (green). **(B)** Vaccine-specific T-cell responses over the course of vaccination. FACS ICS readout values were transformed into pooled ASI values for each analyzed time point and peptide. Each peptide-specific graph depicts CD4+ responses on the left and CD8+ responses on the right. Ticks on the x-axes indicate the patient’s visits according to the vaccination protocol and follow-up schedule; only selected time points are labeled in the bottom row as representative examples of the immunomonitoring timeline: From left to right: V4, V5, V6, V9, V12, V13, V14, V15, V16 (1st Vaccination); T1, T2, V17, V18, V19, T6, T7, T8, T9, T10 (while V17-V19 encompass the second Vaccination cycle). Y-axes ticks show pooled ASI values (logarithmic scale), with a dotted horizontal line at pooled ASI value = 1 (no difference between peptide-stimulated sample and negative control samples) and a bold horizontal line at pooled ASI = 2 indicating at least a twofold increase relative to the negative control. The bottom column “S” represents responses to the WT control peptide (Survivin). Horizontal bars denote treatment phases: first vaccination cycle (green), CPI therapy (orange), and second vaccination cycle (gray). Vertical lines indicate complete remission (CR, green) and fourth recurrence (red). Example FACS plots show DMSO vs. peptide-stimulated IFN-γ and TNF-α double positive T-cells. **(C)** FACS-based evaluation of T-cell clones (P5-7, P3-6, P4-4) specific for peptide 3. Example FACS plots show DMSO vs. peptide-stimulated IFN-γ and TNF-α double-positive T-cells. **(D)** Functional validation of the clonally expanded TCR (P4-4/P5-7), re-expressed in a Jurkat-based triple-reporter cell line using orthotopic TCR replacement (OTR). Reporter activation upon stimulation with HLA-A*02:01^+^ antigen-presenting cells pulsed with the vaccine peptide (purple) or an A2-restricted control peptide (blue-green). Overlay of NFκB (x-axis) and NFAT (y-axis) reporter signals illustrates selective activation in response to the vaccine peptide.


Blood samples collected before each vaccination and throughout the schedule were stimulated for 12 days with vaccine peptides. ICS for IFN-γ, TNF-α, CD154, and IL-2, combined with flow cytometry, was performed to assess CD4^+^ and CD8^+^ T-cell subsets, gated within viable, single, CD56^-^ lymphocytes. Lymphocytes were first identified by FSC/SSC, singlets defined by FSC-A vs. FSC-H, and T-cells were gated as CD3^+^ before separation into CD4^+^ and CD8^+^ subsets. FMO and unstimulated (DMSO) controls were used to set thresholds, and PMA/Ionomycin and viral recall antigens served as positive controls. Simultaneous expression of multiple markers was determined by Boolean gating. Representative gating, functional readouts over time, and clone-specific responses are shown in **Figure 2A-C**. The Stimulation Index (pooled ASI) served to define peptide-specific responses.

$$pooled\;ASI=\frac{\left(SI\;2^{+}antigen\;stim\right)+\left(SI\;3^{+}antigen\;stim\right)+\left(SI\;4^{+}antigen\;stim\right)}{\left(SI\;2^{+}negative\;ctrl\right)+\left(SI\;3^{+}negative\;ctrl\right)+\left(SI\;4^{+}negative\;ctrl\right)}$$

A pooled ASI ≥ 2, indicating a twofold increase in multifunctional T-cells over background, defined a vaccine-specific response at any post-vaccination time point.

The sample schedule encompassed the first cycle (V1-V16), post-vaccination time points T1 and T2, a second cycle (V17-V19), followed by T3-T7. Pre-treatment immunomonitoring revealed no detectable vaccine-specific T-cell responses.

A first CD8^+^ response to peptide 3, characterized by IFN-γ and TNF-α expression, became detectable at V16. Robust CD8^+^ responses reappeared at V17 and V18 and were confirmed at later follow-up samples (T3, T4 and T6). CD8^+^ responses to peptide 4 were first observed at T3, and persisted through T7, whereas responses to peptide 5 were detected between as early as T1 and persisted at T4 -T6. A CD8^+^ response to Survivin was noted at V16. CD4^+^-responses, marked by CD154 and TNF-α, were first observed for peptide 5 at V18 and reappeared at T2 and T4. After initiation of CPI, a CD4^+^ response to Survivin emerged at V15 and persisted at V16, T1 and V17-V19, with a further signal at T4.

These responses persisted beyond the fourth relapse and during follow-up, as confirmed in 2019, 2022, and 2023 (T5-T7). Despite initially low immune signals, the combination of vaccination and Nivolumab amplified immune responses, correlating with tumor regression and durable remission.

T-cell clones specific to peptide 3 were generated by IFN-γ Secretion Assay and FACS-based sorting. Expanded clones underwent peptide restimulation and ICS, confirming specificity. Bulk TCRαβ sequencing revealed identical TCRs (CDR3α CAMSVSSNDYKLSF; CDR3β CASSQLTGGINYGYTF) in two out of three cultures, supporting a clonal CD8^+^ T-cell response. The clonally expanded TCR (P4-4/P5-7) and an additional candidate (P3-6) were re-expressed in a Jurkat-based triple-reporter cell line using a CRISPR-Cas9-mediated orthotopic TCR replacement (OTR) as described by Schober et al. (14). Upon peptide stimulation, only the clonally detected TCR elicited a distinct reporter signal, confirming antigen-specific activation and supporting its functional relevance (**Figure 2D**).

## Discussion

This case highlights the clinical and immunological efficacy of combining neoantigen-targeting peptide vaccination with CPI in a pediatric patient with metastatic HCC. The vaccine successfully induced an *in vivo* T-cell response, contributing to the regression of an inoperable metastasis and long-term disease control throughout a 14-year clinical course. Despite the patient’s immunocompromised state, the treatment was well tolerated and free of severe side effects, underscoring its feasibility in challenging clinical scenarios.

Despite recent advances in the treatment of metastatic HCC, including the combination of checkpoint inhibitors with VEGF-targeting agents, prognosis remains poor, with median overall survival ranging between 12 and 20 months (15, 16). Against this background, the clinical course observed in this case — ongoing complete remission for now over five years without maintenance therapy and despite multiple prior recurrences - represents a highly exceptional outcome.

This long-term recurrence-free remission highlights the potential of long-lived, vaccine-induced T-cell responses to mediate durable tumor control. Moreover, this observation underscores that personalized peptide vaccination can establish effective and lasting antitumor immunity, particularly when integrated into a broader multimodal strategy.

Sequential administration of the peptide vaccine and CPI induced CD4^+^ and CD8^+^ T-cell responses specific to multiple peptides, including peptides 3–5 and Survivin, correlating with tumor regression and durable remission. Immunomonitoring indicated that vaccine-induced T-cells targeted tumor-specific mutations and were further activated by CPI, likely contributing to the observed clinical response and, thus, supporting the synergistic potential of this combination therapy.

Supporting evidence for this synergistic effect comes from the immunomonitoring data: during the initial vaccination cycle up to V15 no tumor-specific T-cell response was detectable (pooled ASI< 2). At V16 in April 2017, shortly after the initiation of Nivolumab in March 2017, a first low-level CD8^+^ response to peptide 3 was observed. Following this, immune responses amplified and diversified, with robust CD8^+^ activity against peptide 3 during V17 and V18. Durable vaccine-specific CD8^+^ responses to peptide 3 were subsequently confirmed at follow-up samples T3, T4, and T6, before declining at T7. Additional CD8^+^/CD4^+^ responses to peptides 4–5 and Survivin further illustrated the breadth of vaccine-induced immunity. These immunological developments correlated with a marked reduction in tumor activity: while FDG-PET-MRI in April 2017 still showed persistent metabolic activity, imaging from October 2017 onwards confirmed complete remission. Based on these findings, we conclude that this interplay between CPI–mediated T-cell enhancement and tumor-specific vaccination contributed to the exceptionally durable remission observed.

During follow-up, T-cell responses were regularly monitored to guide potential booster vaccinations. A second prophylactic cycle was initiated but discontinued due to an allergic reaction, possibly linked to GM-CSF or peptide-specific IgE antibodies. Such reactions, though rare, are typically manageable with corticosteroids, whereas Sargramostim-associated side effects occur more frequently.

The relative contributions of peptide vaccination, CPI, or their combination to tumor regression and durable remission remain difficult to delineate. Previous treatments such as platinum-based chemotherapy, sorafenib and photon radiotherapy had failed to induce durable responses, as evidenced by repeated relapses. Up to V15, no vaccine-specific T-cell response was detectable; at V16, shortly after initiation of Nivolumab, a first low-level response became apparent, which broadened and intensified thereafter, with robust CD4^+^ and CD8^+^ responses emerging during subsequent samples. The close temporal association of these immune responses with radiological regression strongly suggests that the durable remission was attributable to the combinatorial immunotherapy rather than prior conventional treatments. Recurrence after initial remission underscores the importance of investigating resistance mechanisms. Immunohistochemistry of the fourth recurrent lesion revealed 15% PD-L1 expression, suggesting immune evasion through PD-L1-mediated T-cell suppression. While Nivolumab disrupts this interaction, persistent antigen-specific stimulation may promote T-cell exhaustion, potentially contributing to relapse. Mechanisms such as immune escape and antigen evolution – where tumor cells adapt to immune surveillance by altering antigen expression – may further complicate treatment efficacy and highlight the need for continuous monitoring and adaptable treatment strategies.

Compared to other neoantigen-targeting approaches, the presented vaccine targeted a limited number of mutations through HLA class I- and II-restricted peptides. The selected targets encompassed tumor-associated antigens with diverse biological functions – such as intestinal differentiation (P1), JAK/STAT signaling and extracellular matrix modulation (P2), glucose metabolism (P3), cell adhesion and tumor suppression (P4), and an immunogenic but non-oncogenic epitope (P5). This functional diversity supports the rationale for including biologically distinct epitopes in personalized vaccine design. The growing integration of exome sequencing and mutational profiling into clinical practice paves the way for personalized treatment approaches across malignancies. However, peptide vaccines alone may be insufficient in solid tumors, advocating the necessity of combination strategies, particularly with CPIs, to enhance therapeutic efficacy.

Beyond target selection, the clinical benefit of neoantigen vaccination hinges on the quality of the immune response — particularly the durability and antigen specificity of vaccine-induced T-cells. Sustained vaccine-specific CD4^+^ and CD8^+^ T-cell responses were associated with durable tumor control, further supporting the long-term potential of personalized peptide vaccination. The prolonged remission, supported by persistent vaccine-specific T-cells, suggests a potential maintenance effect, reinforcing vaccination as a long-term strategy. These findings emphasize the intricate interplay between immune responses, tumor evasion mechanisms, and therapeutic interventions, highlighting the need to refine combination strategies to optimize treatment outcomes.

This case has several limitations. Due to limited availability of patient material, we could not assess additional exhaustion markers such as TIM-3, LAG-3, or TOX to gain further insights into the dynamics of T-cell exhaustion and potential reactivation under CPI. As a single case study, generalizability to the broader pediatric or adult HCC population remains limited, requiring larger prospective cohorts to validate our findings. In this context, preclinical or early clinical studies have begun to explore peptide vaccination in combination with CPI (17). In a mouse model of HCC, Yang et al. showed that the combination of neoantigen vaccination and anti-PD-1 application resulted in a response rate of approximately 70–80% (18). This was a significant improvement compared to monotherapy groups, where response rates were generally below 40%. Importantly, this combination therapy led to increased CD8^+^ T-cell infiltration, decreased regulatory T-cells, and reduced exhaustion markers on CD8^+^ T-cells, suggesting enhanced anti-tumor immunity. A phase I clinical trial plans to evaluate a peptide vaccine based on the DNAJB1-PRKACA fusion transcript, a characteristic alteration in fibrolamellar HCC, combined with CPI (17). Priority directions in future prospective clinical trials should include the incorporation of prospective immunomonitoring to track vaccine-induced immune dynamics, and adaptive trial designs that allow refinement of vaccine composition based on emerging immunogenicity data. Such strategies could enhance both the clinical efficacy and the translational value of combinatorial immunotherapy in rare liver tumors. Our case, in particular, illustrates that even in advanced-stage pediatric HCC, sustained disease control may be achievable through antigen-specific vaccination strategies, particularly when embedded in a multimodal treatment framework that includes surgery and checkpoint inhibition.

## Data Availability

The original contributions presented in the study are included in the article. Further inquiries can be directed to the corresponding author.
